# Peri-implantation estradiol level has no effect on pregnancy outcome in vitro fertilization- embryo transfer

**DOI:** 10.3389/fendo.2024.1326098

**Published:** 2024-02-12

**Authors:** Pinxiu Huang, Yuan Ou, Ni Tang, Jing Chen, Qiuyue Wen, Jingjing Li, Dingyuan Zeng

**Affiliations:** ^1^ Center of Reproductive Medicine, Guangzhou Women and Children’s Medical Center-Liuzhou Hospital, Liuzhou, Guangxi, China; ^2^ Guangxi Clinical Research Center for Obstetrics and Gynecology, Liuzhou, Guangxi, China; ^3^ Center of Reproductive Medicine, Liuzhou Maternal and Child Health Hospital, Liuzhou, Guangxi, China; ^4^ Center of Reproductive Medicine, Liuzhou Institute of Reproduction and Genetics, Liuzhou, Guangxi, China; ^5^ Affiliated Maternity Hospital and Affiliated Children’s Hospital of Guangxi, University of Science and Technology, Liuzhou, Guangxi, China

**Keywords:** estrogen, luteal phase, IVF-ET, peri-implantation period, pregnancy outcome

## Abstract

**Background:**

The necessity of monitoring luteal endocrine functions in in vitro fertilization- embryo transfer (IVF-ET) remains uncertain. Specifically, the significance of luteal phase estradiol (E2) levels is a matter of debate in current literature.

**Objective:**

To assess the impact of luteal phase (day 11 after HCG trigger) estradiol levels on IVF-ET outcomes.

**Design:**

Twelve thousand five hundred and thirty-five (n = 12,535) IVF-ET cycles performed in our center between 2015 and 2021 were divided into 5 groups based on the middle and late luteal phase serum E2 (MllPSE2) level percentiles as follows: Group A < 50 pg/mL (N=500), group B 50 pg/mL≤E2<150 pg/mL (N=2545), group C 150 pg/mL≤E2<250 pg/mL (N=1327), group D 250 pg/mL≤E2<500 pg/mL (N=925), group E E2≥500 pg/mL (n=668). The clinical pregnancy rates, abortion rates, and live birth rates of each group were compared. Binary logistic regression analysis was carried out to assess the potential impact of MllPSE2 on the live birth rate (LBR).

**Results:**

No significant differences were found in various parameters when comparing the five groups. The level of MllPSE2 showed no significant difference between the pregnant group and the non-pregnant group. The binary logistic regression analysis model demonstrated that MllPSE2 was not significantly related to LBR.

**Conclusion:**

The influence of E2 during the peri-implantation period (day 11) on clinical outcome in IVF-ET is not affected, even if E2<50 pg/mL. It is speculated that ovarian-derived E2 in MllPSE2 is not deemed necessary for endometrial receptivity. Although caution is warranted due to the retrospective nature of the analysis and the potential for unmeasured confounding, it is argued that the need for luteal E2 monitoring in IVF-ET may be of questionable value.

## 1 Introduction

The endometrium undergoes changes in response to fluctuations in ovarian hormonal levels. Within the menstrual cycle, there exists a brief critical phase known as the “implantation window,” during which the endometrium becomes receptive to embryo implantation (1). Elevated progesterone plays a pivotal role in inducing this receptive state (2). Adequate levels of estrogen are also conducive to enhancing endometrial receptivity (3). However, determining the optimal estrogen range, either too low or too high, that might adversely affect human endometrial receptivity has proven challenging. During in vitro fertilization (IVF) cycles, the inhibition of GnRH-a or GnRH-ant on the pituitary gland, coupled with the removal of a substantial number of granulosa cells during oocyte retrieval, results in luteal dysfunction. This, in turn, diminishes the production of estrogen and progesterone, preventing the occurrence of a second peak of these hormones (4). Consequently, several studies have suggested that supplementing with estrogen at this stage can have favorable effects on clinical outcomes (5–7). However, as the importance of luteal support in IVF has become more apparent, other studies have indicated that augmenting luteal support with estrogen may not be advantageous for clinical outcomes (8, 9). Some research has proposed that middle and late luteal phase serum E2 (MllPSE2) levels can serve as predictive indicators of clinical outcomes. Nevertheless, conflicting evidence exists (10–14), with certain studies failing to substantiate these claims (15–17). This controversy highlights the uncertainty surrounding the impact of MllPSE2 on clinical outcomes. Since pregnancy occurrence is influenced by multiple critical factors, including patient age, endometrial thickness, and the quantity and quality of transferred embryos, our aim is to comprehensively assess the influence of MllPSE2 on clinical outcomes through multifactorial analysis. This evaluation seeks to determine the utility of MllPSE2 as a guide for clinical decision-making.

## 2 Materials and methods

### 2.1 Study population

For this retrospective analysis, data were collected from January 2015 to October 2021, encompassing a total of 12,535 cycles of infertility patients who were subjected to IVF-ET treatment at Liuzhou Maternal and Child Health Hospital’s Reproductive Medicine Center in Guangxi. Blood samples for E2 measurement were taken 11 days after HCG trigger.

Inclusion Criteria: 1) The luteal phase GnRH agonist long scheme or the GnRH antagonist scheme was administered. 2) Fresh embryo transplantation was performed, involving at least one excellent-quality embryo. 3) Endometrial thickness exceeded 7mm. Exclusion Criteria: 1) Abnormal uterine anatomy. 2) The presence of endometrial polyps. 3) Hydrosalpinx. 4) Adenomyosis. 5) A high risk of Ovarian Hyperstimulation Syndrome (OHSS). 6) Preimplantation Genetic Testing (PGT), and other specific conditions. After the application of these criteria, a total of 5,965 cycles met the inclusion criteria. These cycles were subsequently classified into five groups based on estrogen levels measured 9 days after oocyte retrieval: Group A: E2 < 50 (N=500) Group B: 50 ≤ E2 < 150 (N=2545) Group C: 150 ≤ E2 < 250 (N=1327) Group D: 250 ≤ E2 < 500 (N=925) Group E: E2 ≥ 500 (N=668).

### 2.2 Ovulation promotion scheme

GnRH agonist long scheme: in the prior middle luteal phase, 1.875 mg/d of GnRH-a (Triptorelin Acetate, Ipsen France Biotechnology Company) was administered. After 20 days, the selection of recombinant follicle-stimulating hormone (rFSH, Gonafen, Serrano Company, Switzerland) was determined based on factors such as patient age, the number of antral follicles, basic hormone levels, and previous ovarian response. Subsequent adjustments to the gonadotrophin (Gn) dosage were made according to follicular size and hormonal fluctuations.

GnRH antagonist scheme: rFSH was initiated on the 2nd or 3rd day of the menstrual cycle. The selection of 75-300 IU of rFSH was based on patient age, the number of basal antral follicles, and basic hormone levels. Adjustments to rFSH dosage were made in response to follicular size and hormonal changes. GnRH-ant (Sizekai, Serrano, Switzerland) was introduced when follicles reached a diameter of 12~16mm, with a daily intramuscular injection of 0.25mg until the date of human chorionic gonadotropin (HCG) administration.

The injection of hCG (human chorionic gonadotropin) from Zhuhai Lizhu Company in China was administered as follows: when 2-3 follicles reached a diameter of ≥18 mm, hCG was injected into the muscle to induce ovulation. After 34-36 hours, oocyte retrieval was performed under transvaginal ultrasound guidance. Depending on the circumstances, either conventional IVF or ICSI was used for fertilization. Embryo transfer took place on the 3rd-5th day following oocyte retrieval.

### 2.3 luteal phase support scheme

On the day of oocyte retrieval, dydrogesterone tablets (10mg, three times a day) from Abbott Biologicals B.V. were taken orally, and micronized progesterone (Utrogestan R, 200 mg, twice daily) was administered vaginally. Between the 3rd and 5th day after egg retrieval, 1-2 embryos were transplanted. Support for the corpus luteum continued until the 12th-14th day after transplantation, with further decisions made based on the pregnancy status.

### 2.4 Observation indicators

Comparison was made regarding the clinical pregnancy rate, abortion rate, and live birth rate. Clinical pregnancy was confirmed by a B-ultrasound examination 35 days post-transplantation. Abortion before 12 weeks of pregnancy was defined as such. Clinical pregnancy rate = (number of clinical pregnancy cycles/total transplantation cycles) × 100%; abortion rate = (number of abortion cycles/number of clinical pregnancy cycles) × 100%; live birth rate = (number of live birth cycles/total number of transplantation cycles) × 100%; Multiple birth rate= number of twins/triplets/number of clinical pregnancy cycles) × 100%.

### 2.5 Statistical analysis

Statistical analysis was performed using SPSS 13.0 software. Statistical evaluation was performed with the Student’s t test, χ^2^ test, Fischer’s exact test, and Variance Analysis (ANOVA), where appropriate. Differences were considered significant at P<0.05.LBR was the main outcome of the study. Binary logistic regression analysis was performed to assess the potential effect of various E2 levels of MllPSE2 adjusting for the following potential confounders: age, foundation FSH, foundation E2, endometrial thickness, eT count, whether high-quality embryos, whether blastocyst, HCG day E2 level. Adjusted odds ratios (aOR) and 95% confidence intervals (CI) were calculated.

## 3 Result

### 3.1 study population

**Figure 1:**
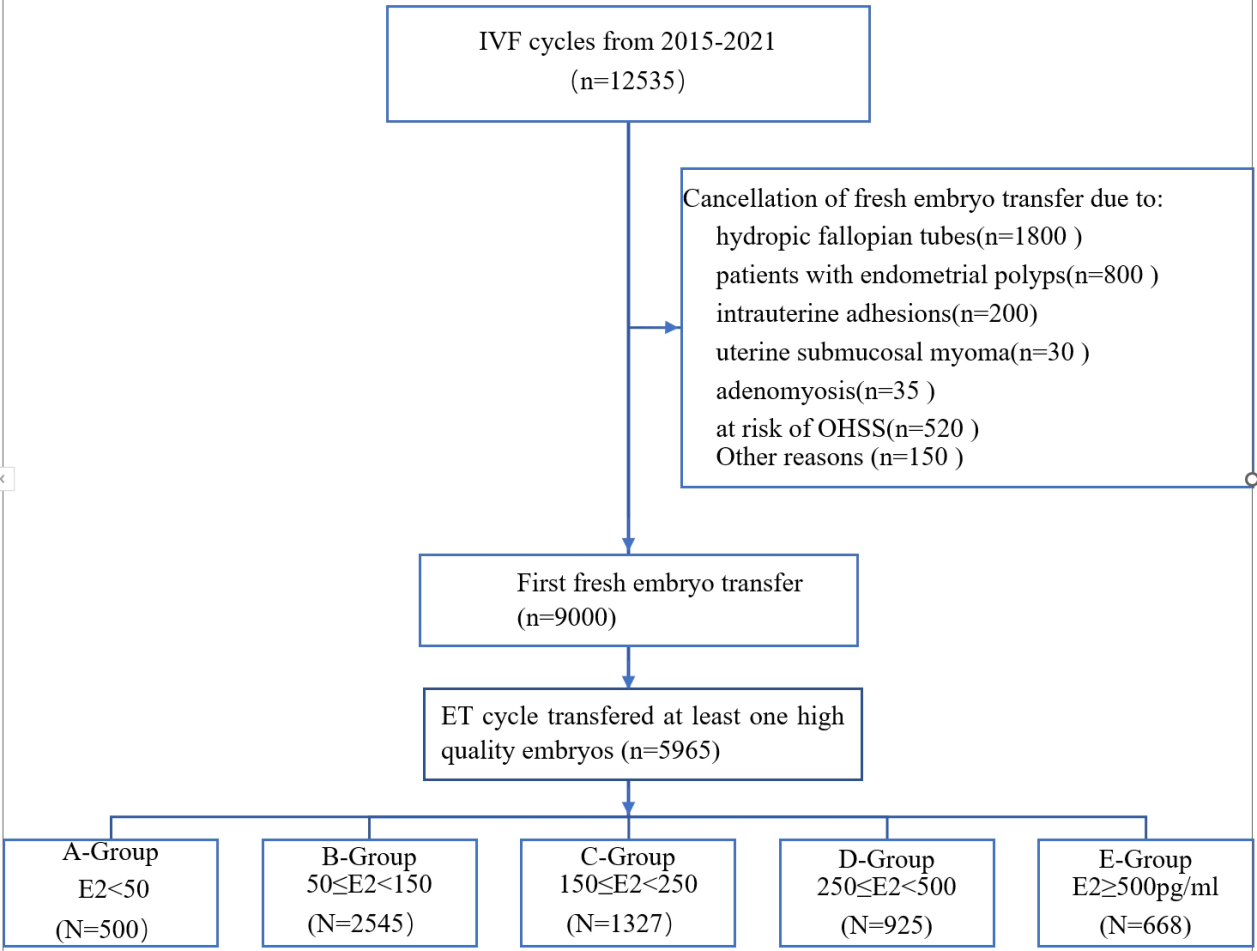
Outline of the selection process in this study.

As depicted in **Figure 1**, out of a total of 12,535 cycles, 3,535 cycles were initially excluded due to various reasons, including fallopian tube volume issues (n=1,800), endometrial polyps (n=800), uterine cavity adhesions (n=200), submucous myoma of the uterine cavity (n=30), adenomyosis (n=35), high OHSS (Ovarian Hyperstimulation Syndrome) (n=520), and other factors (n=150). This left us with 9,000 cycles for fresh embryo transfers. Subsequently, 3,035 cycles without high-quality embryo transfers were excluded from the initial 9,000 cycles. Ultimately, 5,969 cycles met the inclusion criteria. Based on different estrogen levels following oocyte retrieval, they were categorized into five groups: Group A < 50 (N=500), Group B 50 ≤ E2 < 150 (N=2,545), Group C 150 ≤ E2 < 250 (N=1,327), Group D 250 ≤ E2 < 500 (N=925), and Group E E2 ≥ 500 (N=668).

### 3.2 General clinical data of the study population

The means ( ± SD) of various clinical parameters of all the patients included in the study are presented in **Table 1**. The age and basal FSH,E2 and LH level were analyzed in five different group by ANOVA. There were no significant differences observed in age and basal LH. While there were statistical differences in basal FSH and basal E2 levels among the groups, but they remained within the normal range. Refer to **Table 1** for details.

**Table 1:** General clinical data for each group.

	A group E2<50 N=(500)	B group 50≤E2<150 N=(2545)	C group 150≤E2<250 N=(1327)	D group 250≤E2<500 N=(925)	E group E2≥500pg/mL N=(668)	P
Age(years)	34.18 ± 4.62	34.23 ± 4.75	34.26 ± 4.49	34.26 ± 4.89	34.35 ± 4.70	0.620
Basis FSH(IU/L)	6.49 ± 3.00	6.24 ± 2.93	5.81 ± 2.66	5.89 ± 2.43	5.92 ± 2.24	0.001
Basis LH(IU/L)	3.84 ± 2.87	3.94 ± 2.34	3.55 ± 2.48	3.56 ± 2.54	3.55 ± 2.90	0.060
Basis E2	40.96 ± 32.16	43.62 ± 19.74	36.11 ± 21.53	35.87 ± 17.55	36.67 ± 22.73	0.014
Primary infertility	34.00(170/500)	35.00(891/2545)	35.72(474/1327)	36.00(333/925)	33.23(222/668)	0.765
Secondary infertility	66.00(330/500)	65.00(1654/2545)	64.28(853/1327)	64.00(592/925)	66.77(446/668)	0.765
Tubal factor	67.00(335/500)	66.48(1692/2545)	67.14(891/1327)	64.97(601/925)	65.12(435/668)	0.788
Male factor	15.00(75/500)	14.00(356/2545)	15.98(212/1327)	16.00(148/925)	15.42(103/668)	0.633
Ovulation failure	10.00(50/500)	10.00(254/2545)	8.89(118/1327)	11.03(102/925)	9.58(64/668)	0.570
Unexplained infertility and other factors	8.00(40/500)	9.54(243/2545)	7.69(106/1327)	8.00(74/925)	9.88(66/668)	0.297

There were no significant differences observed in primary infertility/secondary infertility ratio, composition ratio of various infertility factors analyzed in five groups (Fischer’s exact test).

### 3.3 Clinical outcomes of IVF in the study population

There were no significant differences observed in the endometrial thickness on HCG day and the number of ET embryos analyzed in five different groups (ANOVA).

No significant differences were found in the antagonist/long regimen ratio, the composition ratio of D3 embryos and D5 day blastocysts, the clinical pregnancy rate, Multiple birth rate, abortion rate, and live birth rate analyzed in five groups (Fischer’s exact test). Refer to **Table 2** for details.

**Table 2:** Clinical outcomes for each group.

	A group E2<50 N=(500)	B group 50≤E2<150 N=(2545)	C group 150≤E2<250 N=(1327)	D group 250≤E2<500 N=(925)	E group E2≥500pg/mL N=(668)	P
Antagonist/long regimen	54.32%(176/324)	53.87%(891/1654)	52.18%(455/872)	52.89%(320/605)	52.51%(230/438)	0.919
HCG endometrium(mm)	11.75 ± 5.51	11.36 ± 3.03	11.46 ± 4.13	11.35 ± 3.15	11.17 ± 3.35	0.17
ET embryo number	1.63 ± 0.50	1.60 ± 0.53	1.58 ± 0.50	1.56 ± 0.50	1.54 ± 0.50	0.96
D3 embryo ratio	30.00%(150/500)	27.98%(712/2545)	26.98%(358/1327)	26.49%(245/925)	25.00%(167/668)	0.335
D5 blastocyst ratio	70.00%(350/500)	72.02%(1833/2545)	73.02%(969/1327)	73.51%(680/925)	75.00%(501/668)	0.335
Clinical pregnancy rate	55.20%(276/500)	57.01%(1451/2545)	57.72%(766/1327)	56.54%(523/925)	55.09%(368/668)	0.765
Multiple birth rate	4.71%(13/276)	4.96%(72/1451)	5.22%(40/766)	5.16%(27/523)	5.43%(20/368)	0.993
abortion rate	15.94%(44/276)	15.99%(232/1451)	14.10%(108/766)	14.53%(76/523)	16.03%(59/368)	0.762
live birth rate	46.40%(232/500)	47.90%(1219/2545)	49.59%(658/1327)	48.32%(447/925)	46.26%(309/668)	0.608

### 3.4 Levels of E2 in secretory period of pregnancy and non-pregnancy

There were no significant differences in age and E2 levels during the secretory period of pregnancy and non-pregnancy analyzed in two groups (Student’s t test). Refer to **Table 3** for details.

**Table 3:** E2 levels during the secretory period in pregnancy and non-pregnancy.

	pregnancy N=(3386)	No pregnancy N=(2579)	P
Age(years)	34.23 ± 4.54	34.35 ± 4.68	0.08
Level of E2 in secretory phase(pg/mol)	150.65 ± 125.26	145.64 ± 120.45	0.07

### 3.5 Binary regression analysis

**Table 4** presents the aOR (adjusted Odds Ratios) with the corresponding 95% CI (Confidence Intervals) and p-values for each parameter included in the regression model. LBR was the main outcome of the study. Age, foundation FSH and E2, endometrial thickness, eT count, whether high-quality embryos, whether blastocyst, HCG day E2 level, various E2 levels of MllPSE2 were incorporated binary regression analysis. The only significant p-value (0.023) was identified for the embryo quality score of the best transferred embryo (aOR 0.321, CI 0.132–0.853). After adjusting no significant differences were observed among the various E2 levels of MllPSE2. (**Table 4**).

**Table 4:** Binary regression model.

		significance	Exp(B)	The 95% confidence interval for the EXP (B)	
				lower limit	upper limit
age		.615	.889	.901	1.110
foundation FSH		.661	.923	.832	1.221
foundation E2		.595	0.986	.896	1.234
endometrial thickness		.228	1.111	.966	1.132
ET count		.499	1.445	.523	3.887
Whether high-quality embryos (1)		.023	.321	.132	.853
Whether blastocyst		.585	1.311	.491	3.551
HCG day E2 level					
E2<50		Reference			
50≤E2<150		0.753	1.015	0.782	2.653
150≤E2<250		0.892	1.323	0.856	2.986
250≤E2<500		0.876	1.123	0.678	3.067
E2≥500pg/mL		0.967	0.943	0.769	2.134
constant		.385	.243		

## 4 Discussion

Progesterone is crucial during the luteal phase, while luteal estradiol may only have a permissive role in relation to the endometrium (2). This study demonstrates that having estradiol levels below 50 pg/mL in the middle and late luteal phase does not impact clinical outcomes. This reaffirms this perspective.

The adverse impact of a significant decline in mid-luteal estradiol levels on implantation is a subject of debate and controversy. In a retrospective analysis of 106 IVF-ET cycles, Sharara and McClamrock (1999) observed that when the estradiol ratio (day of hCG/8 days after hCG) was 5 (indicating an 80% decline), implantation and pregnancy rates significantly decreased. These patients were treated with long or flare-up protocols for controlled ovarian hyperstimulation (COH), with luteal support involving intramuscular progesterone (50–100 mg/d). It has been suggested that this phenomenon might compromise uterine receptivity (18), but only a few recent studies have addressed this issue (19). However, Hung Yu Ng et al. (2000) reported no adverse effects on the outcomes of 763 ART cycles, despite observing a mid-luteal estradiol decline. In this study, all patients received long pituitary downregulation using a daily dose of GnRH-a, and luteal support included either 1,500 IU of hCG on the day of embryo transfer and 6 days later, or intramuscular progesterone (50 mg/d) or vaginal progesterone pessaries (400 mg twice daily). Even when the ratio of day-of-hCG estradiol to mid-luteal estradiol was greater than 5, it had no significant impact on pregnancy rates (20).

In a study by Shevach (2003), a total of 100 patients aged 38 years, including normal- and high-responding patients, had their morning blood collected on days 0 (hCG day), 9, and 14 in a GnRH-agonist scheme. In this study, all patients were administered micronized progesterone (Utrogestan; Basins Iscovesco (C.T.S), Paris, France; vaginal tablets, 100 mg three times daily) for luteal support. Patients receiving hCG as luteal support were excluded from the study. Shevach found that the occurrence of conception did not correlate with the absolute mid-luteal estradiol level or with the 95% percent estradiol decline (D0/D9) in good and high responders (15). Hung Yu Ng et al. (2000) also reported that the mid-luteal estradiol level did not significantly influence pregnancy and implantation rates (20). Furthermore, Laufer (1982) showed that mid-luteal levels of progesterone and estradiol were essentially similar in non-fertile and fertile cycles in non-assisted reproductive technology women (16). In a meta-analysis by Huang (2015), fifteen relevant randomized controlled trials (RCTs) were identified, including a total of 2,406 patients. This meta-analysis suggests that adding estradiol during the luteal phase through oral medication does not improve IVF/ICSI outcomes, even with different daily doses (21). Another meta-analysis by Gelbaya (2008) included ten RCTs that met the criteria for inclusion, and it also showed that adding estradiol to progesterone for luteal phase support in IVF/ICSI cycles does not have a beneficial effect on pregnancy rates (9). Therefore, it is speculated that monitoring blood luteal phase estradiol levels may have limited significance for clinical guidance.

In this study, the mid-luteal phase was defined as 11 days after the trigger date of HCG (referred to as HCG day 11). The E2 levels on HCG day 11 were found not to affect the clinical outcome in IVF-ET. However, there are studies with contrasting conclusions to this one. For instance, in a study by Akihisa (2002), they analyzed the pregnancy rate of 436 women undergoing their first IVF cycles using a long protocol and luteal support with progesterone alone. They found that the pregnancy rate in women with low late-midluteal estradiol levels (measured 7 days after embryo transfer) (< 100 pg/mL) was significantly lower compared to those with medium (100-500 pg/mL) and high (> 500 pg/mL) levels (22). Another study by Ashalatha Ganesh (2009) compared the luteal phase hormonal profile in pregnant and non-pregnant women who underwent mid-luteal long-protocol down-regulation with GnRH-a. They found that Day-7 (7 days after embryo transfer) and Day-14 (14 days after embryo transfer) luteal estradiol levels were significantly different between the two groups (10). Similarly, Florêncio (2008) reached a similar conclusion, observing that estrogen levels in pregnant groups of all ages were significantly higher than those in non-pregnant groups at 7 days after ET (14).

The reason for the inconsistency between these three studies and the conclusion of the current study is that the estrogen levels in the secretory phase in these three studies were measured 7 days after embryo transfer, which is equivalent to 12 days after the trigger date of HCG (HCG day 12). Jin Song et al. (2007) found that there was no correlation between estrogen levels from 2 to 8 days after oocyte retrieval and clinical pregnancy by continuously monitoring estrogen changes at 2, 4, 6, 8, and 10 days after oocyte retrieval. They pointed out that estrogen began to rise 10 days after oocyte retrieval (HCG day 12), suggesting a likelihood of pregnancy (23). Muashe’s (1984) research included 175 cycles using only the HMG/HCG protocol and measured estrogen levels every other day until 13 days after oocyte retrieval. They found that the estrogen level in the pregnant group was similar to that in the non-pregnant group within 0-9 days after oocyte retrieval, but the estrogen level in the pregnant group increased significantly after 11 days following oocyte retrieval, showing a statistically significant difference (17). Both the studies by Jin Song and S. Muashe demonstrated that there was no significant correlation between estrogen levels and pregnancy rates from 0-9 days after oocyte retrieval, but a difference in E2 levels between pregnant and non-pregnant groups appeared only on HCG day 12. In other words, there is no significant difference in estrogen levels between pregnancy and non-pregnancy from HCG day 0 to HCG day 11. However, from HCG day 12 onwards, the estrogen level in pregnancy is significantly higher than that in non-pregnancy. In this study, the mid-luteal phase was defined as 11 days after the trigger date of HCG, unlike the studies by Akihisa, Ashalatha, and Florêncio, which referred to the 12th HCG day. This discrepancy can explain why the conclusions of this study differ from those of Akihisa, Ashalatha, and Florêncio, and indirectly underscores the reliability of the results of this research.

Regarding the impact of estrogen on endometrial receptivity, estrogen during the secretory phase plays a vital role in the implantation of mouse embryos (24). Without estrogen, mouse uteri remain unreceptive, and the endometrium remains dormant, preventing embryo implantation. The reintroduction of estrogen reactivates endometrial receptivity, enabling embryo re-implantation (25). A high dose of estrogen (10ng) shortens the implantation window in mice, whereas a low dose (3ng) prolongs it more effectively than a high dose. However, when estrogen levels drop below 1.5ng, the endometrium only reaches an early acceptance stage (24). Humans and mice share similarities in certain physiological processes during embryo implantation. Thus, it is hypothesized that excessively low or high estrogen levels during human secretion may hinder embryo implantation. However, determining the precise range of detrimental estrogen levels during human embryo implantation is challenging. It’s worth noting that the mechanism of mouse embryo implantation differs from that of humans. Furthermore, rhesus monkeys, closely related to humans, can still conceive and give birth even in the absence of ovarian-derived estrogen during the luteal phase (26). In the context of freezing and thawing cycles, the artificial cycle is utilized to prepare the endometrium, and its clinical pregnancy rate is comparable to that of the natural cycle. It’s important to understand that, despite lacking the second peak of estrogen observed in the natural cycle, the artificial cycle doesn’t impair embryo implantation. In fact, certain studies indicate that endometrial preparation through the artificial cycle during the freeze-thaw cycle shows no correlation between serum E2 levels and the clinical pregnancy rate on the day of transfer (27, 28). In a study by Mackens, 1,222 artificial FETs were categorized into three groups based on late-proliferative serum E2 levels: ≤p10 (E2 ≤144 pg/mL; n = 124), p11–p90 (E2 from 145 to 438 pg/mL; n = 977), and >p90 (E2 >439 pg/mL; n = 121). Their findings revealed no association between late-proliferative phase serum E2 levels and the clinical pregnancy rate following FET in artificially prepared cycles (28). This suggests that even when E2 levels are below 10pg/mL, it does not adversely impact the pregnancy rate, indicating that ovarian-derived estrogen is not essential for endometrial receptivity during the mid-secretory phase.

Although this study is a retrospective analysis with potential confounding factors, such as immunological abnormalities, smoking, previous obstetric complications, and family history. It raises doubts about the clinical value of monitoring E2 levels on HCG day 11 to guide decisions, such as whether to add estrogen support to the corpus luteum or cancel the cycle. This underscores the need to differentiate between the effects of estrogen and progesterone on human endometrial receptivity.

## Data availability statement

The raw data supporting the conclusions of this article will be made available by the authors, without undue reservation.

## Ethics statement

The studies involving humans were approved by Ethics Committee of Liuzhou Maternal and Child Health Hospital. The studies were conducted in accordance with the local legislation and institutional requirements. Written informed consent for participation was not required from the participants or the participants’ legal guardians/next of kin in accordance with the national legislation and institutional requirements.

## Author contributions

PH: Conceptualization, Writing – review & editing, Writing – original draft. YO: Writing – review & editing, Writing – original draft. NT: Data curation, Formal Analysis, Writing – review & editing. JC: Investigation, Methodology, Resources, Writing – review & editing. QW: Investigation, Methodology, Resources, Writing – review & editing. JL: Project administration, Writing – review & editing. DZ: Project administration, Writing – review & editing.
